# Spirostanol Sapogenins and Saponins from *Convallaria majalis* L. Structural Characterization by 2D NMR, Theoretical GIAO DFT Calculations and Molecular Modeling

**DOI:** 10.3390/molecules26102999

**Published:** 2021-05-18

**Authors:** Karolina Dąbrowska-Balcerzak, Jadwiga Nartowska, Iwona Wawer, Paweł Siudem, Katarzyna Paradowska

**Affiliations:** 1 Department of Physical Chemistry, Faculty of Pharmacy, Medical University of Warsaw, Banacha 1, 02-097 Warsaw, Poland; dabrowskakarolina@interia.pl (K.D.-B.); iwona.wawer@wum.edu.pl (I.W.); katarzyna.paradowska@wum.edu.pl (K.P.); 2 Department of Pharmacognosy, Faculty of Pharmacy, Medical University of Warsaw, Banacha 1, 02-097 Warsaw, Poland; jadwiga.nartowska@wum.edu.pl

**Keywords:** *Convallaria majalis*, *Liliaceae*, steroidal sapogenins, spirostanol sapogenins, NMR, GIAO DFT, docking

## Abstract

Two new spirostanol sapogenins (5β-spirost-25(27)-en-1β,2β,3β,5β-tetrol **3** and its 25,27-dihydro derivative, (25S)-spirostan-1β,2β,3β,5β-tetrol **4**) and four new saponins were isolated from the roots and rhizomes of *Convallaria majalis* L. together with known sapogenins (isolated from *Liliaceae*): 5β-spirost-25(27)-en-1β,3β-diol **1**, (25S)-spirostan-1β,3β-diol **2**, 5β-spirost-25(27)-en-1β,3β,4β,5β-tetrol **5**, (25S)-spirostan-1β,3β,4β,5β-tetrol **6**, 5β-spirost-25(27)-en-1β,2β,3β,4β,5β-pentol **7** and (25S)-spirostan-1β,2β,3β,4β,5β-pentol **8**. New steroidal saponins were found to be pentahydroxy 5-*O*-glycosides; 5β-spirost-25(27)-en-1β,2β,3β,4β,5β-pentol 5-*O*-β-galactopyranoside **9**, 5β-spirost-25(27)-en-1β,2β,3β,4β,5β-pentol 5-*O*-β-arabinonoside **11**, 5β-(25S)-spirostan-1β,2β,3β,4β,5β-pentol 5-*O*-galactoside **10** and 5β-(25S)-spirostan-1β,2β,3β,4β,5β-pentol 5-*O*-arabinoside **12** were isolated for the first time. The structures of those compounds were determined by NMR spectroscopy, including 2D COSY, HMBC, HSQC, NOESY, ROESY experiments, theoretical calculations of shielding constants by GIAO DFT, and mass spectrometry (FAB/LSI HR MS). An attempt was made to test biological activity, particularly as potential chemotherapeutic agents, using in silico methods. A set of 12 compounds was docked to the PDB structures of HER2 receptor and tubulin. The results indicated that diols have a higher affinity to the analyzed targets than tetrols and pentols. Two compounds (25S)-spirosten-1β,3β-diol **1** and 5β-spirost-25(27)-en-1β,2β,3β,4β,5β-pentol 5-*O*-galactoside **9** were selected for further evaluation of biological activity.

## 1. Introduction

*Convallaria majalis* from the family *Liliaceae* (lily of the valley) is widely distributed in Europe whereas *Convallaria keisukei* grows in East Asia. *C. majalis* is a known source of cardiac glycosides. However, non-cardiac substances, such as steroidal saponins, are also of pharmaceutical interest [1]. Steroidal saponins have hemolytic, insecticidal, antiparasitic, antifungal, antibacterial, antiviral, anti-inflammatory, antihyperlipidemic, antidiabetic and antitumor properties, among others [2]. Additionally, steroidal glycosides have a wide variety of commercial uses such as surfactants, foaming agents and precursors for the industrial production of pharmaceutical drugs [3].

Convallasaponins were isolated from the flowers of *C. keisukei* [4,5,6,7,8,9] and from the roots of *C. majalis* [10,11]. Tschesche and co-workers isolated convallamaroside, a 22-hydroxyfurostanol saponin with three sugar chains (at C-27, C-3 and C-1); by its partial hydrolysis and cyclization of the side chain, the convallamaronin was formed, of which, the aglycone is convallamarogenin, identified in 1973 as Δ25-5β,20β,22α-spirosten-1β,3β-diol **1**. Besides convallamaroside, other steroidal saponins (13 compounds) were isolated from the roots and rhizomes of *C. majalis* by means of column chromatography [12], however, at that time the compounds could not be characterized by 2D NMR spectroscopic methods. The 1β,3β,4β,5β-tetrol was isolated from *C. keisukei* and named convallagenin-B **6** [5], its ^13^C NMR spectra were recorded in 1981 [13]. The pentahydroxy sapogenin (5β-spirost-25(27)-en-1β,2β,3β,4β,5β-pentol **7** (pentologenin) and (25S)-spirostan-1β,2β,3β,4β,5β-pentol 5-*O*-β-d-glucopyranoside **10** (neopentologenin 5-*O*-glucoside) were found in *Aspidistra elatior* (*Liliaceae*) [14,15] and pentologenin was characterized by NMR [16]. Further investigation on the steroidal glycosides of the rhizomes of *C. majalis* resulted [17] in the isolation of a 5β-spirostanol triglycoside, named convallasaponin A (elucidated as (25S)-3β,17α-dihydroxy-5β-spirostan-1β-yl *O*-β-d-glucopyranosyl-(1→2)-*O*-[β-d-xylopyranosyl-(1→3)]-β-d-quinovopyranoside), along with two known cardenolide glycosides and a cholestane glycoside.

As described, the saponin compounds found in *C. keisukei* have been well studied and described. The aim of our work was to determine the content of saponin compounds in the European variety *C. majalis*, and then to study their biological activity.

Saponins exhibit various biological properties; they act as cytotoxics inducing apoptosis in tumor cells and inhibiting tumor-induced angiogenesis. So far, there have been few reports on biological activity associated with the exposure to particular saponins of *C. majalis*. The triglycoside convallasaponin A exhibited potent cytotoxic activity against human submandibular gland carcinoma (HSG) cells [17]. The convallamaroside was found to be an anti-angiogenic compound [18] with immunotropic properties [19].

The investigation of biologically active substances, such as steroidal saponins, isolated from plant resources, is one of the most important issues of the chemistry of natural and physiologically active compounds. Structural studies are the first step that leads to bioactivity research, including molecular modeling methods. Molecular modeling helps to better understand the binding mode of target compounds at the molecular level. Nowadays, they are commonly used as preliminary investigations before in vitro and in vivo biological studies.

The studies using in vitro and in vivo models have been conducted to evaluate the anticancer activity of various steroidal saponins. The reports suggest that saponins exhibit antiproliferative and apoptotic activity acting by means of several molecular mechanisms.

Human epidermal growth factor receptor 2 (HER2) is expressed in many tissues and its major role is to facilitate excessive cell growth and tumorigenesis. HER2 is one of the most interesting targets in addressing intracellular signaling pathways in cancers. [20].

The introduction of HER2 directed therapies has significantly influenced the outcome of patients with HER2 positive breast and gastric cancers [21].

Tubulins are targets for anticancer drugs such as paclitaxel, and the vinca alkaloids, which are known to bind to human tubulin [22]. Therefore, a set of 12 isolated from *C. majalis* compounds was docked to the structures of HER2 receptor and tubulin to evaluate their potential activity.

**Figure 1:**
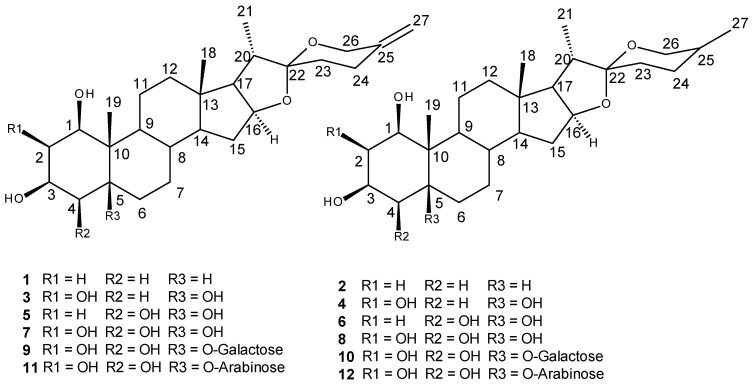
Structures of sapogenins and saponins isolated from *Convallaria majalis* described in the present study with carbon numeration.

The isolation of tetra- and pentahydroxy 5β-spirostanes and their glycosides from *C. majalis* was first reported in 2005 [23]. As a continuation of our studies on the structure and biological properties of the constituents of *C. majalis* we presently report the isolation of eight sapogenins (1–8) and four saponins (9–12) (Figure 1) and their characterization by NMR spectroscopy supported by theoretical calculations (GIAO DFT).

## 2. Results

The powdered roots and rhizomes of *C. majalis* were macerated and extracted with methanol/water (*v*:*v* 1:1). After concentration, a crude extract of saponins was separated into fractions: CHCl_3_ (I) and *n*-BuOH + H_2_O (II). The *n*-BuOH fraction was subjected to column chromatography after completing acid hydrolysis. The isolation yielded eight crystalline sapogenins, two 1,3-diols: **1**, which turned out to be convallamarogenin and **2** dihydroconvallamarogenin, according to [11], two 1,2,3,5-tetrols: **3** and **4**, two 1,3,4,5-tetrols: **5** and **6**, as well as two sapogenins with five hydroxy groups **7** and its dihydro-derivative **8**. Four new glycosides, the 5-*O*-galactosides **9** and **10** and 5-*O*-arabinosides **11** and **12** were isolated directly from (I) and the convallamaroside from (II).

It is worth noting that a pair of compounds with spirosten or spirostan moiety, such as **1** and **2**, is probably present in the plant naturally. Yet, it cannot be excluded that spirostanol-type sapogenin came from the cyclization of the original glycoside with a furost-26-ol moiety during extraction and separation procedures. The chromatographic separation of steroid sapogenins bearing the same number of OH groups and separation of neo-and iso-isomers (with a different configuration of the C-25 methyl group) is difficult in a mixture [24]. Tschesche et al. [25] reported in 1961 that **1** convallamarogenin and **2** dihydroconvallamarogenin are characterized by the same R_f_ values (0.50) in TLC, and dihydromarogenin was obtained by catalytic hydrogenation of convallamarogenin. Similarly, it was not easy to perform chromatographic isolation of pure spirostens or spirostans, which were present as admixtures in small quantities (of a few percent). Therefore, pure dihydro-derivatives (such as **4**) were obtained by hydrogenation of the respective spirostens (on PtO_2_, for 2 days, at room temperature).

Compounds **1**–**12** were obtained as colorless crystals; unfortunately, our attempts to prepare single crystals suitable for X-ray diffraction studies were unsuccessful.

The LSI mass spectrum of **1** was consistent with the molecular formula C_27_H_42_O_4_, MS of **2** showed an [M + H]^+^ ion at *m*/*z* = 433, which is higher than that of **1** by two mass units. The presence of two more hydrogens was suggestive of a saturated fragment (spirostan) and molecular formula C_27_H_44_O_4_. The melting points and the values of [α]D are in accordance with the data given by Tschesche et al. [25] for convallamarogenin **1** and dihydroconvallamarogenin **2**.

The ^1^H NMR spectrum of **1** in CDCl_3_ contained two steroid methyl proton signals at δ 0.77 (s, H-18), 1.26 (s, H-19) and a doublet at δ 0.95 (H-21) (Table 1). The 2D HETCOR spectra allowed the identification of quaternary carbons (C-10, 13, 22), directly coupled with ^1^H-^13^C pairs and axial/equatorial hydrogens of most methylene groups. The ambiguities, due to the number of protons with very similar chemical shifts (particularly in the region δ1.1 to 2.1 ppm) could be resolved by observation of the connectivities in the COSY and HMBC spectra. The ^1^H and ^13^C signals of **2** were observed essentially in the same positions as those of **1**, except for the signals due to the E- and F-ring (Table 2).

^13^C NMR (δ109.4 of C-22 and δ16.0 of C-27, see Table 3) of **2** indicated that it was a 25S spirostanol derivative, in agreement with the negative [α]_D_ value of −30° [11]. The cross-peaks between C-22 and H-26 signals in the HMBC spectrum confirm the presence of ring F at C-22 (i.e., it is spirost-25(27)-en, not the furost-25(27)-en structure). The (25S)-spirosten-1β,3β-diol 1 has not been characterized yet by ^1^H and ^13^C NMR spectroscopy, whereas ^13^C NMR spectrum of (25S)-spirostan-1β,3β-diol **2** (25,27-dihydro-convallamarogenin, also named rhodeasapogenin) was assigned by Tori et al. [13].

The LSI MS of **3** showed an [M + H]^+^ ion at *m*/*z* = 463 indicating that molecular weight should be 462 and molecular formula: C_27_H_42_O_6_. The number of oxygen atoms, besides oxygens in the furan and spirostan rings, suggested that this compound should have four OH groups. In the ^1^H NMR spectra the H-27 signal appeared at 4.76/4.79 ppm, and the signals of three methyl groups: Me-18, Me-19 and Me-21 (doublet) can be observed. The ^13^C NMR spectrum of **3** included 27 intense signals: 4× CH_3_, 9× CH_2_ and 10× CH, according to DEPT experiment; standard ^13^C spectra showed a double bond between C-25 (δ142.9) and C-27 (δ108.2). The order of methyl carbons was unusual since C-19 resonance appeared at 12.3 ppm, whereas in the spectra of **1** and **2**—at 18.9 ppm. A strong shielding effect (6.6 ppm) resulted from nearby β-hydroxyl groups [26,27]. The stereochemistry at C-5 and other carbons of ring A was confirmed by the NOESY spectrum. The cross-peaks H-1/H-11, H-2/H-9 or H-9/H-4 indicate the *cis*-fusion of the A/B rings. Using the ^1^H-^13^C correlation (HETCOR), the signals which appeared between 65 and 77 ppm were assigned to the carbons bearing the OH groups. The configuration of hydroxyl groups at C-1 and C-3 was determined to be β (axial) from the multiplicity of the H-1 (a broad singlet), H-2 (triplet-like, 3J < 3.5 Hz) and H-3 (a broad singlet). The strong cross peak in the NOESY spectrum between H-2 and H-9α shows that H-2 is on the α-side. The cross peak H-9α and H-4 at 2.39 ppm (and the absence of the peak H-9α/H-4 δ1.85) can be understood assuming a reverse assignment of the H-4 axial/equatorial pair, since H-4α, axial and H-9α are proximal.

The MS of **4** showed *m*/*z* = 465, indicating a compound of molecular formula C_27_H_44_O_6_. The ^1^H NMR spectrum in CDCl_3_ + CD_3_OD (1:1) exhibited two three-proton singlet signals at δ 0.79 (H-18) and 1.25 (H-19) and two three-proton doublet signals at δ 1.00 (H-21) and 1.09 (H-27), which are characteristic of a spirostanol derivative.

Inspection of NMR data shows that ^1^H and ^13^C chemical shifts for **3** and **4** are the same for the A, B and C rings (Table 1 and Table 3); this is also true of other spirosten/spirostan pairs. On the other hand, spirostans exhibit similar spectra within fragments assigned to the D, E and F rings, especially the pattern of long-range correlations in the HMBC spectra. These correlations (Table 3) are sufficient to identify the spirostanol structure of the F-ring.

The 25S configuration of **4** can be deduced from the analysis of ^13^C chemical shifts of C-25 to C-27. These values are in agreement with assignments of the F-ring carbon signals of 25S-spirostans reported earlier [13,28].

The MS data of **5** and **6** suggested that those compounds should also have four OH groups and an [M + H]^+^ ion at *m*/*z* = 463 and 465, thus indicating that their molecular formulas should be C_27_H_42_O_6_ and C_27_H_44_O_6_, respectively. The ^1^H spectrum of **5** exhibited a characteristic doublet at 4.78/4.81 ppm corresponding to olefinic protons of the = CH_2_ group and three signals of methyl groups: singlets of angular Me-18, Me-19, and one doublet of Me-21. The signals at 143.8 ppm (C25) and 108.9 ppm (C27) in the ^13^C spectrum confirmed the 25(27)-spirosten structure. However, the analysis of NMR spectra clearly showed that the hydroxylation pattern was different from that of **3** or **4**. The connectivities observed in the ^1^H-^1^H COSY and ^1^H-^13^C HMBC spectra allowed the identification of four carbons bearing hydroxyl groups as C1, C3, C4 and C5 whereas C2 was a methylene carbon. Thus, the structure of **5** was determined as 5β-spirost-25(27)-en-1β,3β,4β,5β-tetrol.

The ^13^C NMR spectrum of **6** was very similar to that of **5** except the signals due to the F-ring (C22–C27, see Table 4). The resonance of Me-27 (3H doublet in the ^1^H spectrum at 10.6 ppm) at 15.9 ppm and the resonances of C23 and C24 at ca. 26 ppm evidenced 25S-spirostanol configuration. Compound **6** was found to be 25S-spirostan-1β,3β,4β,5β-tetrol, identical with convallagenin-B (III) isolated from *C. keisukei* [5].

Mass spectrometry data suggested that the molecular formula of **7** and **8** was higher by one oxygen atom than that of **3**–**6**. The ^1^H spectrum of **7** showed three signals of methyl groups (Me-18, Me-19 and Me-21) and four signals of H1–H4 from methine groups. In the ^13^C spectra five signals of oxygenated carbons (including quaternary one at δ 78.7) were observed between 67 and 80 ppm. This suggested that there are five hydroxyl groups in the A-ring part. Characteristic signals of a spirost-25(27)-en at δ144.2 (C25) and 108.9 (C27) and the analysis of COSY and HMBC correlations indicated that the F-ring does not have a furostane-type structure. Compound **7** was finally assigned as 5β-spirost-25(27)-en-1β,2β,3β,4β,5β-pentol; previously isolated from the underground part of *Aspidistra elatior* BLUME (*Liliaceae*) by Hirai [14].

Unambiguous assignments of ^1^H and ^13^C NMR signals of **8** were made using COSY and HMBC correlations (Table 2). Chemical shifts of the A-E rings are the same as for **7**, except those belonging to the F-ring. The ^1^H spectrum showed four methyl signals, including a doublet of Me-27. In the HMBC spectrum carbon signals at δ26.6 (C24), 28.2 (C25) and 65.6 (C26) were correlated with Me-27. ^13^C chemical shifts of C-22 (δ110.5) and C-27 (δ16.5) showed clearly that it was a 25S spirostanol derivative. Comparing the NMR data of **8** with those of a known compound neopentologenin, the ^13^C chemical shifts were found to be identical or very similar, suggesting that they had the same configuration of the F-ring (Table 4). On the basis of those data, **8** was identified as (25S)-spirostan-1β,2β,3β,4β,5β-pentol; it was isolated from *Aspidistra elatior* by Konishi [15].

The fraction (I) which was subjected to a series of chromatographic separations without hydrolysis gave compounds **9**–**12**. The NMR spectra of those saponins were measured in pyridine-d5 to minimize the signal overlap. The molecular formula of compound **9** was determined as C_33_H_52_O_12_ by the pseudo-molecular peak at *m*/*z* = 663.3 [M + Na]^+^ in the positive FAB MS. Acid hydrolysis and TLC analysis confirmed that the sugar was galactose. The ^1^H NMR spectrum showed signals for two angular methyl groups at δ 0.82 ppm and 1.72 ppm (singlets), a doublet at 1.07 ppm (Me-21), and one anomeric proton signal at δ 5.23 ppm, suggesting **9** to be a spirostanol glycoside. The signals of four oxygenated carbons appear in the range 67–78 ppm and that of C5, linked to sugar, at 88 ppm (Table 5). This means that the aglycone is pentahydroxylated spirostanol **7**. In the HMBC spectrum, the correlation between the anomeric proton of galactose and the quaternary carbon signal at 87.9 ppm showed that the sugar was linked to C5. The signal of C5 was shifted by 9.2 ppm downfield and the C1, C4, C6 carbon signals were shifted by 0.7–5.4 ppm upfield in comparison with those of **7**, in agreement with the glycosidation shifts. The ^1^H and ^13^C NMR data of sugar moiety were similar to the data determined previously [29].

Compound **10** was identified to be (25S)-pentahydroxyspirostanol 5-β-d-galactoside. The FAB-MS showed an [M + Na]^+^ ion at 665.3; for C_33_H_54_O_12_Na. The ^1^H NMR spectrum showed signals for two angular methyl groups, two doublets (Me-21 and Me-27) and one anomeric proton signal. The COSY, HMBC and NOESY correlations confirmed the spirostanol structure of the F-ring and penta-hydroxylation of the A-ring.

The molecular formula of **11** was established as C_32_H_50_O_11_ by FAB-MS (*m*/*z* 633.3 [M + Na]^+^). Acid hydrolysis yielded arabinose and a genin, identified as **8**. A cross peak between H1 (δ 5.23 ppm) of arabinose and the quaternary C5 signal (δ 86.9) in the HMBC spectrum provided definite evidence for an ester linkage. Interpretation of NMR spectra and comparison with the reported data [23] confirmed the pentahydroxyspirosten structure.

Saponin **12** was assigned the molecular formula C_32_H_52_O_11_ by FAB-MS ([M + Na]^+^ at *m*/*z* 635.3). The ^1^H and ^13^C NMR data (Table 1 and Table 5) demonstrated that the aglycon of **12** was pentahydroxyspirostanol, similar to that of **10**, but bearing a pentose unit. Its five signals observed in the ^13^C NMR spectrum corresponded to arabinose, and in the HMBC spectrum the long-range correlations of the anomeric proton signal with the respective carbon signals (C5, C4, C6) showed that the structure can be formulated as (25S)-spirostanol 5β-spirostan-1β,2β,3β,4β,5β-pentol 5-*O*-β-arabinonoside or neopentologenin 5-*O*-β-arabinonoside **12**.

The chemical shift of C6 is informative–this signal appeared at 34.3 ppm in compounds with methylene carbon C4 (**3** and **4**), and the substitution with 4β-OH results in a ca. 4 ppm upfield shift (δ30.4 in **7**, **8**); in saponins **9–12** with sugar linked to C5 a further increase in shielding occurs (δ24.4–25.0) due to the steric hindrance.

Preliminary investigations of biological activity using in silico methods were then performed. Due to potential antiproliferative activity of saponins, two targets were chosen to verify possible mechanisms: HER2 receptor and tubulin. A set of 12 ligands was docked to the PDB structures. Based on the docking results, the compounds were ranked by comparing total score values and each received RANK value according to this order (Table 6).

## 3. Discussion

Compounds **1**–**12** isolated from *C. majalis* are all polyhydroxylated steroidal saponins with 5β-H, 5β-OH or 5β-*O*-sugar as the common structural feature.

Steroidal saponins with sugar moiety attached to the angular position of C5 were first found in *C. keisukei* by Kimura et al. in 1968 (convallasaponin-B, formulated as convallagenin-B α-l-arabinopyranoside). It is worth noting that in a series of studies on saponins of Japanese *Convallaria keisukei* no spirost-25(27)-ens were reported. The spirostanol-type sapogenins with two, three and four hydroxyl groups at ring A were isolated from both *C. keisukei* and *C. majalis* (Table 3). The negative Cotton effects were consistent with the A/B-*cis* ring fusion, and the S configuration at C-25 [30]. All hydroxyl groups, including 5β-OH, were considered to have β-configuration.

The convallasapogenin-A was elucidated as 25S-5β-spirostan-1β,3β,5β-triol [5], one of the few 25S trihydroxysapogenins, contrary to the 25R series, more frequently isolated from natural plant sources. The tetrols and their hydroxylation pattern are also interesting. Kogagenin ((25R)-spirostan-1β,2β,3β,5β-tetrol) isolated in 1959 from *Dioscorea tocoro* [31] was the first example of a naturally occurring spirostan tetrol; its ^13^C NMR data were published in 1981 [13]. However, no corresponding 25S-5β-spirostan derivative was isolated. Convallagenin-B, isolated by Kimura et al. [5] was the first tetrahydroxy steroidal sapogenin with 25S configuration, and can be regarded as a 25S isomer of kitigenin, 25R-spirostan-1β,3β,4β,5β-tetrol. The 25S-5β-spirostan-1β,3β,4β,5β-tetrol **6** is a new representative of tetrol of the 25S series. These tetrols and their dihydro derivatives are unique in having the 1β,3β,4β,5β- and 1β,2β,3β,5β-hydroxylation patterns.

The sapogenin with all hydroxylated carbons of the A-ring, i.e., with 1β,2β,3β,4β,5β-hydroxylation, had already been found in *Liliaceae*. The glycoside, neopentologenin 5-*O*-β-glucopyranoside had been isolated from *Rhodea japonica* (THUNB.) ROTH and *Apidistra elatior* BLUME [15]. This is the first report on the isolation and structural characterization of 5β-spirost-25(27)-en-1β,2β,3β,4β,5β-pentol **7** (neopentologenin) and their glycosides, 5-*O*-β-galactoside **9** and 5-*O*-β-arabinonoside **11**, from *Convallaria majalis*.

The molecules of sapogenins show little conformational freedom, except the reorientation of the OH groups in the A-ring. Theoretical calculations of NMR shielding constants (see Table 3, Table 4 and Table 5) allowed the assignment of all ^13^C resonances in the ^13^C NMR spectra by comparing the experimental chemical shift values with the theoretical NMR parameters. It is possible by converting the shielding constants into chemical shift using the formula: δiso = σTMS-σiso, where the calculated value of the shielding constant relates to the tetramethylsilane shielding constant (TMS). Since the interpretation of the A- and F-ring signals is difficult and ambiguous, GIAO DFT calculations were especially helpful; these data may complement or even replace classic assignments using 2D NMR.

**Figure 2:**
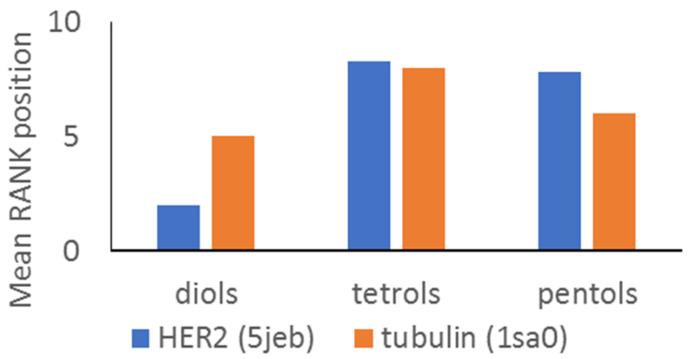
Mean RANK position from molecular docking to HER2 and tubulin for diols, tetrols and pentols.

This chapter deals with investigations of biological activity using in silico methods. Figure 2 illustrates the mean RANK value calculated for diols, tetrols and pentols. An earlier study of steroidal glycosides from *Convallaria majalis* showed that the introduction of polar substituents to the steroidal nuclei resulted in reduced cytotoxicity [32]. Our docking results suggest that diols (less polar) have a higher affinity to the analyzed targets than tetrols and pentols (more polar). However, not only the structures of the aglycone moiety but also the sugar sequences in the steroidal glycosides may affect its affinity. All glycosides were 5-hydroxy substituted, **9** and **11** had the same aglycone moiety as **10** and **12**. Compound **9** (galactoside) had RANK position from docking to HER2 and tubulin 2 and 1, respectively, whereas compound **10** (also galactoside) had 12 and 15 positions. Two arabinosides **11** and **12** have 6 and 2 RANK and 4 and 6 RANK positions, respectively. It shows that the sugar type in glycosides is also related to the binding effect.

Molecular docking shows that compounds **1** and **9** should be selected for further evaluation of biological activity due to their best RANK values for both molecular targets. Inspired by the study of Abd El-kader et al. [33] we analyzed the binding mode of the compounds with HER2 receptor and investigated their similarity to doxycycline.

**Figure 3:**
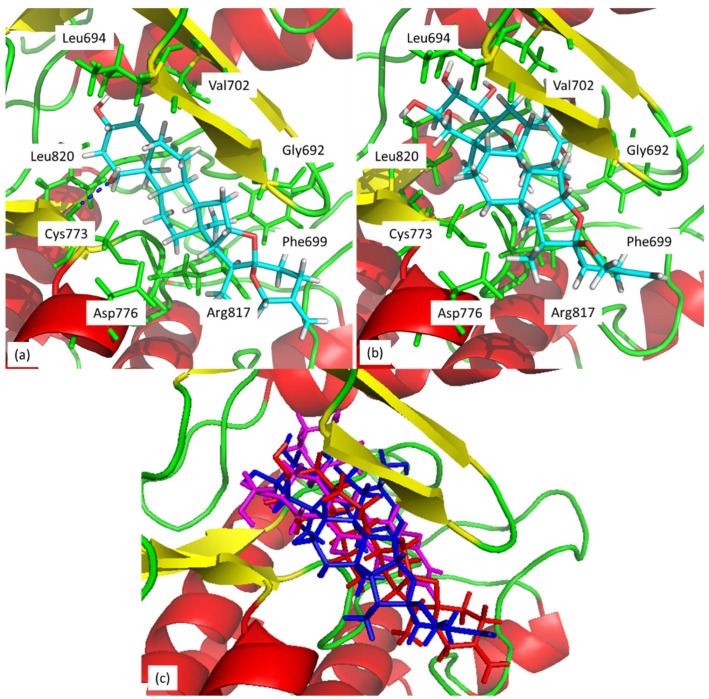
Docking and binding pattern of compounds (**a**) **1**, (**b**) **9**, (**c**) together **1** (red molecule), **9** (blue molecule), and doxycycline (magenta molecule), in the active site of the HER2 receptor.

Compound **1** forms one hydrogen bond with Cys773, whereas **9** does not incorporate H-bond in the active site of HER2 receptor. However, both compounds may show hydrophobic interactions with amino acids residues in the binding pocket, such as Leu694, Val702, Gly692, Phe699, Arg817, Asp776, Leu820 (Figure 3). Our results are in agreement with the results of Abd El-kader et al. [33]. Since their docking results were confirmed by biological experiments, we could expect similar effects. Further evaluation of biological activity of the hydroxylated saponins is in progress.

## 4. Materials and Methods

### 4.1. General Experimental Procedures

Optical rotation was measured using a JASCO P-1020 polarimeter at 20 °C. An AMD-604 mass spectrometer was used for LSI-MS and FAB MS. NMR analysis: ^1^H and ^13^C NMR spectra were recorded on a Bruker DRX-500 spectrometer; the spectra of **1**, **2** in CDCl_3_ and **3**–**12** in CDCl_3_ + CD_3_OD (*v*:*v*, 1:1) solution. The spectra of saponins **8**–**12** were also run for pyridin-d5 solutions. The 2D ^1^H-^13^C correlations were performed using the phase-sensitive gradient-selected HSQC inverse technique; the HMBC experiment was optimized for *J* = 5 Hz. Standard pulse programs from Bruker library were used for ^1^H-^1^H COSY, NOESY and ROESY experiments. The assignment of ^13^C and ^1^H chemical shifts was performed based on 2 D experiments. Chemical shifts were reported in ppm relative to internal TMS.

CC: silica gel 60 (MN) 100–200 mesh, TLC: Kieselgel 60G (Merck). The spots on TLC were visualized by spraying with 10% H_2_SO_4_ or with a mixture of 1% cinnamic aldehyde in EtOH/acetic acid anhydride with conc. H_2_SO_4_. The following solvent systems were used: (A) toluene, (B)–(E) toluene-methanol: (B) (99:1), (C) (95:5), (D) (90:10), (E) (72:28), (F) benzene-ethanol (92:8) and (G) CHCl_3_–MeOH-H_2_O (83.5:15:1.5).

### 4.2. Plant Material

The roots and rhizomes of *C. majalis* were collected in Poland, from the garden of the Agriculture University of Warsaw (SGGW, 51.82° N, 19.90° E). The voucher specimen is deposited at the Department of Pharmacognosy, The Medical University of Warsaw.

Extraction and separation.

The dried rhizomes and roots (700 g) were powdered, macerated with methanol-water (*v*:*v*, 1:1) and extracted twice (1.5 L × 2) for 24 h. The MeOH/water solution was combined, filtered off and concentrated under reduced pressure to 1/3 of its volume. The residue was extracted with CHCl_3_ to give (I) and then with *n*-BuOH + H_2_O (II). The butanolic fraction was evaporated to dryness and the residue (9.6 g) was subjected to acid hydrolysis.

### 4.3. Acid Hydrolysis of the Butanolic Fraction

A solution in 1 M HCl in MeOH (100 mL) was refluxed for 8 h on a boiling water bath. After cooling the reaction mixture was neutralized and submitted to partition into sugar and aglycone fractions.

### 4.4. Isolation of the Sapogenins

The aglycones were separated by CC on a silica gel by using the developing system: (A) to (E) to afford two main fractions, III (200 mg) and IV (311 mg). These fractions finally gave eight compounds: **1** (90 mg), **2** (22 mg) from III, **3** (70 mg). Additionally, **4** (7 mg), **5** (31 mg), **6** (19 mg), **7** (11 mg) and **8** (28 mg) from IV. Larger amounts of **2**, **4** and **6** were obtained by catalytic hydrogenation.

### 4.5. Isolation of Saponins

The procedure of extraction from plant material was repeated and the filtrate of n-butanolic fraction (II) was subjected to CC, and following the procedure described by Tschesche [11,25] the major saponin, convallamaroside was obtained. Steroidal glycosides were separated from (I). After removal of the solvent, the CHCl_3_ + MeOH (9:1) phase was repeatedly chromatographed on silica gel (100–200 mesh, from Merck) with the solvent system: CHCl_3_-MeOH-H_2_O (*v*:*v*:*v*, 90:10:1; 65:35:1) to furnish compounds **9**–**12** using method described elsewhere [9].

### 4.6. Catalytic Hydrogenation

The spirosten (e.g., **3**) was dissolved in MeOH and hydrogenated at room temperature over PtO_2_ for 2 days; the catalyst was removed by filtration and the solution was evaporated to dryness. The presence of spirostane was confirmed by ^13^C NMR spectra (lack of C-25 resonance at δ > 140 ppm).

**1**. 5β-spirost-25(27)-en-1β,3β-diol (convallamarogenin) 

Colorless powder, mp 252–258 °C (lit. mp 258–263 °C, [α]_D_ −78° (CHCl_3_; c 0.92), [25]; ^1^H and ^13^C NMR spectral data (in CDCl_3_), see Table 1, Table 2 and Table 3.

**2**. 5β-(25S)-spirostan-1β,3β-diol (25,27-dihydroconvallamarogenin) 

Colorless powder, mp 274–276 °C (lit. mp 274, [α]_D_ −30° (pyridine; c 0.98), [11,25]; ^1^H and ^13^C NMR spectral data (CDCl_3_) see Table 1 and Table 2, respectively.

**3**. 5β-spirost-25(27)-en-1β,2β,3β,5β-tetrol, (majaligenin) 

Colorless powder, mp 289–295 °C, temp. of sublimation 270 °C, high-resolution positive FAB MS *m*/*z*: calc. for C_27_H_42_O_6_Na: 485.2874, found 485.2885; [α]_D_^25^ –29.65° (CHCl_3_; c 0.23); ^1^H NMR (MeOH) and ^13^C NMR (CDCl_3_), see Table 1, Table 2 and Table 3.

**4**. 5β-(25S)-spirostan-1β,2β,3β,5β-tetrol (25,27-dihydromajaligenin) 

Colorless powder, mp 291–293 °C, high-resolution positive FABMS *m*/*z*: calc. for C_27_H_44_O_6_Na: 487.3030, found 487.3025; [α]_D_^25^ −75.14° (CHCl_3_; c 0.05); ^1^H and ^13^C NMR spectral data (MeOH) see Table 1 and Table 2.

**5**. 5β-spirost-25(27)-en-1β,3β,4β,5β-tetrol, (majaligenin B)

Colorless powder, mp 277–278 °C, [α]^D^ −46.0° (CHCl_3_ + MeOH (*v*:*v*, 1:1); c 0.22), C_27_H_42_O_6_ MS *m*/*z*: 485.3; ^1^H and ^13^C NMR spectral data (MeOH + CDCl_3_) see Table 1 and Table 2.

The known compounds are: 5β-25(S)-spirostan-1β,3β,4β,5β-tetrol, (convallagenin B) **6** [5]; 5β-spirost-25(27)-en-1β,2β,3β,4β,5β-pentol (pentologenin) **7** [14] and 5β-25(S)-spirostan-1β,2β,3β,4β,5β-pentol (neopentologenin) **8** [15].

**9**. 5β-spirost-25(27)-en-1β,2β,3β,4β,5β-pentol 5-O galactoside (pentologenin galactoside)

Colorless powder, mp 258–262 °C, high-resolution positive FABMS *m*/*z*: calc for C_33_H_52_O_12_ Na: 663.3351, found 663.3369; ^1^H and ^13^C NMR spectral data (pyridine-d5), see Table 1 and Table 2, respectively.

**10**. 5β-(25S)-spirostan-1β,2β,3β,4β,5β-pentol 5-*O*-galactoside (neopentologenin galactoside)

Colorless powder, 259–262 °C, high-resolution positive FABMS *m*/*z*: calc for C_33_H_54_O_12_ Na: 665.3507, found 665.3489; ^1^H and ^13^C NMR spectral data (pyridine-d5), see Table 1 and Table 2, respectively.

**11**. 5β-spirost-25(27)-en-1β,2β,3β,4β,5β-pentol 5-*O*-arabinoside (pentologenin arabinoside)

Colorless powder, mp 198–204 °C, high-resolution positive FABMS *m*/*z*: calc for C_32_H_50_O_11_Na: 633.3245, found 633.3250; ^1^H and ^13^C NMR spectral data (pyridine-d5), see Table 1 and Table 2, respectively.

**12**. 5β-(25S)-spirostan-1β,2β,3β,4β,5β-pentol 5-*O*-arabinoside (neopentologenin arabinoside)

Colorless powder, mp 198–203 °C, high-resolution positive FABMS *m*/*z*: calc for C_32_H_52_O_11_Na: 635.3402, found 635.3395; ^1^H NMR spectral data (pyridine-d5), see Table 1 and Table 2, respectively.

### 4.7. Theoretical Calculations

The molecules were first optimized at the PM3 level. Quantum-chemical calculations were carried out using Gaussian 09 software [34]. Further optimizations were performed using the DFT method (B3LYP 6-31G(d,p)). At the same level of theory, vibrational frequencies and intensities were computed. The final low-energy structures with positive vibrational frequencies were used for calculations of NMR shielding constants.

Then, the experimental chemical shift values (δ_iso_) were compared with the theoretical NMR parameters. It was possible by converting the shielding constants (σ_iso_) into chemical shift using the formula: δ_iso_ = σ_TMS_-σ_iso_, where the calculated value of the shielding constant relates to the tetramethylsilane shielding constant (σ_TMS_ = 191.8 ppm) calculated at B3LYP 6-31G(d,p).

Linear regression was determined for the relationship of the experimental chemical shifts and the calculated theoretical chemical shifts. The correlation between the experimental and computational results is presented in the form of the R^2^ correlation coefficient at the bottom of Table 3, Table 4 and Table 5.

The correlation of the calculated chemical shifts with experimental data in each case is satisfactory because R^2^ > 0.99. Therefore, as a second criterion of the quality of calculations was chosen the mean absolute error (MAE) between theoretical and experimental data and enclosed at the bottom of Table 3, Table 4 and Table 5. The combination of the R^2^ coefficient and the MAE error in our previous studies gave satisfactory results in assessing the quality of the theoretically obtained structures in relation to the experimental NMR data [35].

The calculations allowed to verify and confirm the correct assignment of signals based on 2D spectra.

### 4.8. Molecular Docking

The molecular docking of sapogenins and saponins against tubulin protein and HER2 receptor was carried out using Sybyl X 1.2 (Tripos International) software. The structures were drawn in Sybyl-X 1.2 Sketch, then hydrogens were added, and finally, the structures were optimized (Tripos forcefield, gradient 0.05 kcal/mol*A). Molecular target (HER2 receptor and tubulin) was taken from RSC Protein Data Bank (PDB id: 5JEB and 1SA0, respectively). The structures were prepared by removing water, adding hydrogens, and performing the optimization using Sybyl structure preparation tools. Surflex protomols (an idealized active site ligand) were defined based on the ligand position in the crystal structures from PDB. Other parameters of Surflex were used as default values. The best docking poses were chosen according to total score values.

## 5. Conclusions

Steroidal saponins have a wide range of biological properties, besides those known for decades, including antimicrobial, anti-inflammatory and anticancer. New spirostanol sapogenins and saponins were isolated from the roots and rhizomes of *Convallaria majalis* L. New steroidal saponins were found to be tetra- or pentahydroxy 5 β-*O*-glycosides, with all OH groups on the same side of the A ring, bearing arabinose or galactose unit. Theoretical calculations of NMR shielding constants allowed the assignment of all ^13^C resonances in the ^13^C NMR spectra. GIAO DFT calculations were especially helpful; these data may complement or even replace classic assignments using 2D NMR. The molecular activity of different saponins is attributed to their structural composition, e.g., the number of OH groups. Our docking results suggest that diols (less polar) have a higher affinity to the analyzed targets than tetrols and pentols (more polar). The sugar moiety contributes to heteropolarity of saponins, and may lead to different bioactivity.

## Figures and Tables

**Table 1:** ^1^H NMR data (δ in ppm, ref. TMS, J in Hz) for sapogenins **1** and **2** in CDCl_3_, **3**–**8** in CDCl_3_+ CD_3_OD (1:1) and saponins **9**, **11** (in pyridine-d5).

H	1	2	3	4	5	6	9/10 ^a^	11/12 ^b^
1	3.83 br s	3.83 br s	3.86, br s	3.86, br s	3.85	3.85	4.25, br s	4.25, br s
2	1.96; 1.74	1.96; 1.74	3.65, dd (3.3, 3.4),	3.65, dd (3.3,3.4),	2.16; 1.75	2.16; 1.75	4.10, br,s	4.07
3	4.18 br s	4.18 br s	4.16, br s,	4.16, br s	4.14	4.14	4.68, br	4.69, br s
4	2.05, 1.48	2.05, 1.48	2.39 dd (15.6, 3.7); 1.85, d (15.6)	2.39 dd (15.6, 3.7); 1.85, d (15.6)	3.91	3.91	4.35	4.37
5	1.66	1.66	-	-	-		-	-
6	1.46; 1.30	1.46; 1.30	1.74; 1.44	1.74; 1.44	1.95; 1.44	1.95; 1.44	2.72, d, (9.8); 1.85	2.75 d (10.1) 1.95
7	1.95, 1.12	1.95, 1.12	1.41, 1.91	1.41, 1.91	1.58, 1.04	1.58, 1.04	1.33, 1.15	1.35, 1.13
8	2.05	2.05	1.71	1.71	1.70	1.70	1.72	1.70
9	1.23	1.23	1.08	1.08	1.08	1.08	1.26, m	1.21
11	1.30; 1.22	1.30, 1.22	1.45	1.45				1.45
12	1.72; 1.10	1.72; 1.10	1.75; 1.15	1.75; 1.15	1.72; 1.11	1.72; 1.11	1.59; 1.12	1.60; 1.05
14	1.14	1.14	1.13	1.13	1.11	1.11	1.12	1.00
15	2.05; 2.00	2.05 m	1.99 m (16.5); 1.30	1.99 m (16.5); 1.30	1.99; 1.29	1.99; 1.29	2.02; 1.37	1.99, 1.40
16	4.44 dt (16.1, 7.5)	4.43	4.45	4.44	4.45	4.46	4.58, m	4.57m
17	1.77	1.77	1.76	1.76	1.77	1.77	1.80	1.79
18	3H, s, 0.77	3H, s, 0.78	3H, s, 0.81	3H, s, 0.79	0.79	0.79	3H, s, 0.83	3H, s, 0.82
19	3H, s, 1.13	3H, s, 1.12	3H, s, 1.26	3H, s, 1.25	1.32	1.32	3H, s, 1.66	3H, s, 1.72
20	1.89m	1.82m	1.92 m	1.84 m	1.91 m	1.85 m	1.96	1.90, m
21	3H, d (7.0) 0.95	3H, d (7.0) 0.95	3H, d (7.0), 0.96	3H, d (6.9) 0.99	3H, d 0.97	3H, d 0.98	3H, 1.05	3H, d (6.7) 1.07
23	1.27	-	1.74	1.02 d (3.0) 1.63 dt (15.8, 3.0)	1.75; 1.28	1.09	1.80, m	1.80
24	2.56 td (13.5, 5.5) 2.24 d (13.5, 2.2)	-	2.56 td (13.1, 5.5) 2.27 dt (13.5, 2.2)	2.27 (12.6, 2.2)	2.54	1.94; 1.49	2.24 d (12.7)	2.24 d; 2.69
25	-	ca. 1.7	-	1.73 m	-	1.74	-	-
26	4.30 d (12.2) 3.87 d (12.2)	3.94 dd (11.0, 2.6) 3.30 d (11.0)	4.29 d (12.0) 3.85 d (12.0)	3.31 d (11.0) 3.94 dd (11.0, 2.7)	4.25; 3.81	3.91; 3.26	4.47 d 3.36 4.25^10^	4.49 d; 4.04 4.08^12^
27	4.77, 4.74	3H, d (7.0) 1.07	4.79 d (0,9); 4.76	3H, d (7.0) 1.09	4.77	3H, d (7.0) 1.05	4.82, 4.79 4.73, 4.76^10^	4.81, 4.78 1.09^12^

^a^ chemical shifts of Ara: δ 5.23 (^1^H, d, *J* = 5.9 Hz, H-1′), 4.48 (^1^H, H2′, H5′a), 4.38 (^1^H, H-4′), 4.31 (^1^H, H-3′), 3.90 (^1^H, d, *J* = 11.0 Hz, H-5′b). ^b^ chemical shifts of Gal: δ 5.23 (^1^H, d, *J* = 7.8 Hz, H-1′), 4.42 (^1^H, H-2′), 4.41 (^1^H, H-4′), 4.36–4.38 (^1^H, H-6′a,b), 4.20 (^1^H, H-3′), 4.19 (^1^H, H-5′).

**Table 2:** Characteristic correlations in the 2D spectra of **1** and **3**.

1		3	
Long-range correlations in the HMBC spectra			
H-18 (Me) H-19 (Me) H-21 (Me) H-27 H-1 C-3 C-14 C-16 C-17 C-22 C-25	C-12, C-13, C-14, C-17 C-1, C-5, C-9, C-10 C-17, C-20, C-22 C-24, C-25, C-26 C-2, C-3 H-4ax H-15eq, ax, H-9, H-12 H-15eq, H-17, H-23 H-15eq, H-20 H-26ax, eq H-26ax, eq, H-23	H-18 (Me) H-19 (Me) H-21 (Me) H-27 H-1 H-2 H-4eq H-4ax H-15eq H-17 H-26	C-12, C-13, C-14, C-17, C-20 C-1, C-5, C-9, C-10 C-17, C-20, C-22 C-24, C-25, C-26 C-2, C-3, C-5, C-10, C-19 C-1, C-3 C-2, C-3, C-5, C-10 C-5, C-6 C-13, C-14, C-17 C-18 (Me), C-21 (Me) C-22, C25 (δ142.9), C-27
Correlations assigned from COSY spectra			
H-18 (Me) H-21 (Me) H-1 H-3 H-16 H-24eq H-24ax H-27	H-14 H-20 H-2eq, ax H-2eq, ax; H-4 eq,ax H-15 eq,ax H-23, H-24ax, H-27 H-23, H-24eq, H-26ax H-26ax, eq, H-24eq	H-21 (Me) H-27 H-1 H-2 H-3 H-15eq H-16 H-27	H-20, H-23 H26a, b H-2, H-3 H-1, H-3 H-1, H-2, H-4ax, eq H-15ax, H-14 H-15eq, ax, H-17 H-26eq
The “through-space” correlations in the NOESY spectra *			
H-18 (Me) H-19 (Me) H-1 H-9	H-8, H-11 H-8, H-11 H-11, H-19 (Me) H-2, H-4	H-18 (Me) H-19(Me) H-21 (Me) H-1 H-3 H-9	H-8, H-11, H15ax, H-17, H-20 H-1, H-8, H-11ax H-17, H-20, H-23 H-1, H-3, H-11, H-19 H-2, H-4ax, eq H-2, H-4ax (δ2.05)

* Without apparent ones, between geminal hydrogens.

**Table 3:** ^13^C NMR chemical shifts (δ in ppm) for compounds **1**, **2** (sol: CDCl_3_; s.s.: solid state) and **3**, **4**, tetrols 1,2,3,5 (sol: CDCl_3_+ CD_3_OD (1:1); s.s.: solid state) with calculated chemical shifts (δ in ppm) Verification parameters at the bottom of the table: correlation coefficients R^2^ and mean absolute errors MAE.

	1		2		1 and 2	3		4		3 and 4
C Atom No.	δ_C sol_. [ppm]	Calculated δ_C_ [ppm]	δ_C sol_. [ppm]	Calculated δ_C_ [ppm]	δ _C s_. _s_. [ppm]	δ_C sol_. [ppm]	Calculated δ_C_ [ppm]	δ_C sol_. [ppm]	Calculated δ_C_ [ppm]	δ _C s_. _s_. [ppm]
1	73.8	69.7	73.8	69.7	73.5	76.7	76.9	76.7	76.9	79.1
2	32.1	32.4	32.1	32.4	32.2	66.8	67.1	66.8	67.1	66.8
3	68.4	65.5	68.4	65.5	68.3	70.3	71.7	70.3	71.7	71.3
4	33.7	31.8	33.7	31.8	33.8	37.5	40.7	37.5	40.7	36.6
5	30.4	28.0	30.4	28.0	30.5	74.7	75.3	74.7	75.3	76.7
6	26.1	28.0	25.9	28.0	25.9–26.0	34.3	36.8	34.3	36.8	35.6
7	26.0	26.4	25.8	26.4		28.0	31.3	28.0	31.3	28.7
8	35.5	37.3	35.5	37.3	36.4	34.0	36.5	34.0	36.5	34.0
9	41.9	43.7	41.9	43.7	42.2	45.0	46.8	45.0	46.8	46.0
10	39.7	38.5	39.7	38.5	40.0	44.5	43.9	44.5	43.9	46.7
11	20.7	24.9	20.7	24.9	21.3	20.8	24.9	20.8	24.9	23.6
12	40.1	37.6	40.0	37.6	40.8	39.3	36.9	39.3	36.9	41.6
13	40.4	41.0	40.3	41.0	40.8	40.0	41.3	40.0	41.3	42.4
14	56.3	55.8	56.3	55.8	56.5	55.6	55.5	55.6	55.5	56.9
15	31.7	35.0	31.7	35.0	32.2	31.2	35.2	31.1	34.5	31.4
16	81.0	83.3	81.1	83.3	80.9	80.7	83.0	80.5	85.2	82.2
17	62.4	63.1	62.6	63.1	63.6	61.8	63.2	61.7	53.8	58.9
18	16.5	17.4	16.5	17.8	17.2	16.3	17.3	15.8	17.6	19.2
19	18.9	12.8	18.9	15.1	19.5	12.3	12.9	12.3	12.9	15.7
20	41.5	45.1	41.5	45.6	41.9	41.2	45.0	41.7	47.2	41.2
21	14.5	16.3	14.7	16.3	14.5	13.7	16.2	13.5	15.7	16.5
22	109.4	108.9	109.4	109.3	109.6	109.2	109.2	109.7	107.3	110.6
23	32.8	35.3	25.9	30.0	32.0–34.0_(1)_	32.3	35.1	25.4	29.5	32.8_(3)_
24	28.5	27.7	25.7	24.7	25–29	27.9	27.9	25.1	25.0	27.6_(3)_
25	143.6	134.4	27.1	26.3	141.0_(1)_/26.1_(2)_	142.9	134.2	26.6	26.2	145.9_(3)_
26	64.9	63.0	64.7	63.2	65.2	64.5	63.1	64.8	63.0	66.6
27	108.6	103.2	16.0	15.9	108.9_(1)_	108.2	103.3	15.3	15.6	109.5_(3)_
R^2^	0.996		0.995		0.999 *_(1)_	0.998		0.994		0.999 *_(3)_
MAE	2.43		1.91		0.49 *	2.13		2.20		1.58 *

* Corresponds to a comparison of chemical shifts between measurements in solution and in solid state.

**Table 4:** ^13^C NMR chemical shifts (δ in ppm) for compounds **5**, **6** (sol: CDCl_3_+ CD_3_OD (1:1); s.s.: solid state) and **7**, **8** (sol: CDCl_3_+ CD_3_OD (1:1) with the calculated chemical shifts (δ in ppm) Verification parameters at the bottom of the table: correlation coefficients R^2^ and mean absolute errors MAE.

	5		6		5 and 6	7		8	
C Atom No.	δ_C sol_. [ppm]	Calculated δ_C_ [ppm]	δ_C sol_. [ppm]	Calculated δ_C_ [ppm]	δ _C s_. _s_. [ppm]	δ_C sol_. [ppm]	Calculated δ_C_ [ppm]	δ_C sol_. [ppm]	Calculated δ_C_ [ppm]
1	73.9	70.6	73.9	70.6	75.6	78.8	77.6	78.8	77.6
2	32.3	32.2	32.3	32.2	30.0–34.0	67.6	67.0	67.6	67.0
3	70.7	70.1	70.7	70.1	73.8	75.7	76.3	75.7	76.3
4	67.7	67.5	67.7	67.5	69.8	68.5	69.6	68.5	69.6
5	78.5	78.6	78.5	78.6	79.4	78.7	77.4	78.7	77.4
6	29.9	31.4	29.9	31.4	30.7 28.1	30.4	32.1	30.4	32.1
7	28.2	30.1	28.2	30.1		28.9	29.5	28.9	29.5
8	35.0	36.7	35.0	36.7	34.0	35.8	36.2	35.8	36.2
9	45.9	46.6	45.9	46.6	46.8	46.1	46.2	46.1	46.2
10	45.1	43.9	45.1	43.9	46.8	45.6	43.3	45.6	43.3
11	21.3	24.8	21.3	24.8	23.5	22.3	24.8	22.3	24.8
12	40.1	37.1	40.1	37.1	42.7	40.5	37.1	40.5	37.1
13	41.0	41.2	41.0	41.2	42.7	41.5	41.2	41.5	41.2
14	56.4	55.6	56.4	55.6	57.1	56.9	55.6	56.9	55.6
15	31.9	35.2	31.9	35.2	30.0–34.0	32.6	35.2	32.6	35.2
16	81.5	83.2	81.3	83.2	82.2	82.2	83.2	82.2	83.2
17	62.6	63.5	62.3	63.0	64.0	63.6	63.5	63.5	63.0
18	16.5	17.4	16.5	17.6	19.4	17.0	17.4	17.0	17.6
19	13.1	12.8	13.1	12.7	16.1	13.2	12.7	13.2	12.7
20	41.9	45.1	42.4	45.6	42.7	42.8	45.1	43.2	45.6
21	14.5	16.3	14.3	16.3	16.1	14.6	16.3	14.5	16.3
22	109.9	108.9	110.4	109.3	110.3	110.5	108.9	111	109.3
23	33.1	35.3	26.1	30.2	25.0–34.0	33.5	35.3	26.8	30.2
24	28.6	27.7	25.8	24.6		29.0	27.7	26.6	24.6
25	143.6	134.4	27.4	26.4	145.1_(5)_	144.2	134.4	28.2	26.4
26	65.2	63.0	65.2	63.2	66.3	65.6	63.0	66.0	63.2
27	109.0	103.2	16.0	15.9	109.7_(5)_/18.0_(6)_	108.9	103.2	16.5	15.9
R^2^	0.997		0.997		0.999 *_(5)_	0.998		0.997	
MAE	1.93		1.51		1.35 *	1.81		1.46	

* Corresponds to a comparison of chemical shifts between measurements in solution and in solid state.

**Table 5:** ^13^C NMR chemical shifts (δ in ppm) for compounds **9**, **10** pentahydroxy galactosides (sol: pyridine-d5; s.s.: solid state) with the calculated with the calculated chemical shifts (δ in ppm) and **11**, **12** (sol: pyridine-d5). Verification parameters at the bottom of the table: correlation coefficients R^2^ and mean absolute errors MAE.

	9		10		9 and 10	11	12
C Atom No.	δ_C sol_. [ppm]	Calculated δ_C_ [ppm]	δ_C sol_. [ppm]	Calculated δ_C_ [ppm]	δ _C s_. _s_. [ppm]	δ_C sol_. [ppm]	δ_C sol_. [ppm]
1	78.1	77.5	78.1	77.5	79.8	77.2	77.2
2	67.3	67.2	67.3	67.2	67.0	67.1	67.1
3	75.9	76.9	75.9	76.9	75.8	75.6	75.6
4	68.2	69.2	68.2	69.2	68.7	67.6	67.6
5	87.9	83.7	87.9	83.7	93.6	86.9	86.9
6	25.0	30.2	25.0	30.2	27.0 28.7	24.4	24.4
7	28.8	30.4	28.8	30.4		28.1	28.1
8	35.3	36.6	35.3	36.6	34.7	34.2	34.2
9	47.0	47.1	47.0	47.1	46.7	46.2	46.2
10	46.9	44.4	46.9	44.4	46.7	45.8	45.8
11	22.1	25.2	22.1	25.2	23.2	21.2	21.2
12	40.5	37.1	40.5	37.1	40.2	39.5	39.5
13	41.1	41.2	41.1	41.2	42.7	41.0	41.0
14	56.5	55.5	56.5	55.5	57.4	55.6	55.6
15	32.4	35.1	32.4	35.1	32.1	31.7	31.7
16	81.9	83.0	81.9	83.0	83.1	81	80.8
17	63.0	63.1	63.0	63.0	66.4	62.6	62.4
18	16.9	17.5	16.9	17.6	17.1	16.1	16.1
19	13.5	13.1	13.5	12.7	14.0	13.5	13.5
20	42.5	45.1	43.0	45.6	42.8–44.8	42.1	41.5
21	14.6	16.2	14.5	16.3	16.0	14.6	14.4
22	110.5	109.3	110.9	109.3	110.2	109.0	109.3
23	33.5	34.9	26.6	30.2	32.9_(9)_	32.8	26.0
24	29.0	28.0	26.4	24.6	29.5_(9)_	28.6	25.8
25	144.8	134.1	28.0	26.4	146.2_(9)_	144.0	27.1
26	65.5	63.0	65.9	63.2	66.4	64.7	64.6
27	109.1	103.2	16.4	15.9	108.8_(9)_	108.4	15.9
1′	97.3	97.8	97.3	97.8	96.1	97.2	97.2
2′	73.0	76.0	73.0	76.0	73.7	72.8	72.8
3′	74.7	75.1	74.7	75.1	74.2	73.8	73.8
4′	69.8	68.0	69.8	68.0	70.7	68.1	68.1
5′	76.5	75.2	76.5	75.2	78.9	65.8	65.8
6′	61.8	59.3	61.8	59.3	62.5	-	-
R^2^	0.997		0.997		0.999*	-	
MAE	2.01		1.70		1.01*	-	

* Corresponds to a comparison of chemical shifts between measurements in solution and in solid state.

**Table 6:** Compounds **1**–**12** ordered according to their total score function from docking to HER2 and tubulin.

Compound	HER2	Tubulin
	Ranking Position (RANK)	
**1**	1	3
**2**	3	7
**3**	11	4
**4**	7	8
**5**	5	9
**6**	10	11
**7**	8	10
**8**	9	12
**9**	2	1
**10**	12	5
**11**	6	2
**12**	4	6

## Data Availability

The data presented in this study are available on request from the corresponding author.
